# Interplay Between Silicon and Iron Signaling Pathways to Regulate Silicon Transporter *Lsi1* Expression in Rice

**DOI:** 10.3389/fpls.2020.01065

**Published:** 2020-07-22

**Authors:** Nanthana Chaiwong, Nadia Bouain, Chanakan Prom-u-thai, Hatem Rouached

**Affiliations:** ^1^ BPMP, Univ Montpellier, CNRS, INRAE, Montpellier SupAgro, Montpellier, France; ^2^ Agronomy Division, Department of Plant and Soil Sciences, Faculty of Agriculture, Chiang Mai University, Chiang Mai, Thailand; ^3^ Department of Plant, Soil, and Microbial Sciences, Michigan State University, East Lansing, MI, United States; ^4^ Plant Resilience Institute, Michigan State University, East Lansing, MI, United States

**Keywords:** rice, silicon, iron, *Lsi1*, nutrient signaling crosstalk

## Abstract

Silicon (Si) is not an essential element, but it is a beneficial element for growth and development of many plant species. Nevertheless, how plants regulate the initial uptake of silicon (Si) remains poorly understood. It has been proposed that the regulation of Si uptake is largely regulated by Si availability. However, the current model is clearly reductionist and does not consider the availability of essential micro-elements such as iron (Fe). Therefore, the present study investigates the regulation of the Si transporter *Lsi1*, in three rice varieties grown under different Si and Fe regimes. The *Lsi1* transcript was compared to intracellular concentrations of Si and Fe in roots. The amount of *Lsi1* transcript was mainly altered in response to Si-related treatments. Split-root experiments showed that the expression of *Lsi1* is locally and systemically regulated in response to Si signals. Interestingly, the accumulation of *Lsi1* transcripts appeared to be dependent on Fe availability in root growth environment. Results suggest that the expression of *Lsi1* depends on a regulatory network that integrates Si and Fe signals. This response was conserved in the three rice cultivars tested. This finding is the first step toward a better understanding of the co-regulation of Si homeostasis with other essential nutrients in plants. Finally, our data clearly show that a better understanding of Si/Fe signaling is needed to define the fundamental principles supporting plant health and nutrition.

## Introduction

In many plant species, the application of silicon (Si) appears to be beneficial for growth and development (Ma and Yamaji, 2015). Silicon enhances plant resistance against disease, insects, fungi, and abiotic stresses such as drought, nutrient imbalance, toxic metals, and excessive temperatures (Ma and Yamaji, 2008; Guntzer et al., 2012; Zhu and Gong, 2014). In rice, higher Si accumulation was attributed to high Si uptake ability of plant roots, mediated by Si transporters (*Lsi1* and *Lsi2*). It has been demonstrated that *Lsi1* and *Lsi2* are Si transporters during influx and efflux, respectively, while both are localized in root exodermis and endodermis (Ma et al., 2006; Ma et al., 2007). *Lsi1* is responsible for the transport of Si from an external solution to cortical roots cells, whereas *Lsi2* is an efflux transporter responsible for the transport of Si from root cells to the apoplast. These genes are required for the efficient uptake of Si in rice (Ma et al., 2006; Bauer et al., 2011; Deshmukh and Bélanger, 2016). Si accumulation in the shoots of rice plants appears to be controlled by the expression of *OsLsi1* and *OsLsi2* (Mitani-Ueno et al., 2016), while the genes are down-regulated by Si application (Ma et al., 2002; Ma et al., 2006; Ma et al., 2007; Mitani-Ueno et al., 2016).

Recent studies reported that applying Si can increase the uptake of macro and micronutrients, especially in nutrient deficient conditions (Etesami and Jeong, 2018; Soratto et al., 2019). For instance, the beneficial effects of interactions between Si and the essential micronutrient, iron (Fe), on plant growth and production has been reported in many plant species such as cucumber (Gonzalo et al., 2013), soybean (Pavlovic et al., 2013), and barley (Dragama et al., 2019). Applying Si significantly reduced Fe deficiency symptoms in plant. A proposed explanation for this effect includes the improvement of Fe translocation from root to shoot resulting in an increased concentration of Fe in the youngest leaves, maintaining the balance of other micronutrients such as the Fe/Mn ratio and increasing the oxidizing capacity of roots, which changes ferrous iron (Fe^2+^) into ferric iron (Fe^3+)^ (Gonzalo et al., 2013; Pavlovic et al., 2013). Applying Si was also found to alleviate Fe deficiency symptoms in cucumber by increasing shoot and root biomass, chlorophyll content and leaf Fe concentration in Fe deficient plants (Bityutskii et al., 2014). These features are attributed to Si-induced accumulation of Fe mobilizing compounds such as citrate (in leaves and roots) and catechin (in roots) (Bityutskii et al., 2014). Research has also shown that application of Si delayed chlorophyll degradation, decelerated growth reduction, and maintained leaf Fe content under Fe deficiency conditions in soybeans (Gonzalo et al., 2013). It was suggested that the addition of Si increased Fe uptake by up-regulating the expression of genes involved in the reduction based strategy for Fe acquisition in barley plants grown under Fe deficient conditions, resulting in increased Fe content in the youngest leaves (Dragama et al., 2019). This Si-Fe interaction was mainly reported in monocots. For example, in rice plants, applying Si has been shown to improve plant growth by increasing the activity of catalases and polyphenol oxidases in both the root and shoot under Fe deficient conditions (Etesami and Jeong, 2018). The increase in the expression of rice Si transporters after Si application can modulate Fe uptake and translocation by increasing Fe nutrition under deficient conditions (You-Qiang et al., 2012).

As it has yet to be studied, the present research investigates the expression patterns of Si transporter genes and related mechanisms responsible for Si accumulation under Fe deficient conditions. The ways in which *Lsi1* is regulated in response to combinatorial Si and Fe deficiency induced stress in rice plants was studied. We have considered three rice varieties for this study using a split-roots system to dissect between the local from a systemic signal to regulate *Lsi1*. By analyzing the Si and Fe concentrations in conjunction with *Lsi1* relative quantities, it is shown that the expression of Si uptake transporter is regulated by local and systemic Si signals. It is further demonstrate that expression of *Lsi1* was regulated to achieve significant coordination between Si and Fe homeostasis in rice plants. Our results clearly show that while it is important to understand how plants make sense of and adapt to various nutrient signals, gaining knowledge of how plants respond to multiple stresses is equally significant.

## Materials and Methods

### Plant Material and Growth Conditions

Three rice varieties were considered in this study. The two modern popular high yielding rice varieties used in this experiment were Chainat 1 (CNT1) and Pathumthani 1 (PTT1) together with a popular medium yielding variety, Khao Dawk Mali 105 (KDML105). The experiments were conducted in a controlled-environment chamber (light/dark cycle of 14/10 h, 200 µmol photons·m−2·s−1), temperature of 28/25°C, and relative humidity of 80%. Seeds were soaked in deionized water overnight in darkness. Then, seedlings were exposed to light for two days and transferred to 1/4 of the full-strength nutrient solution for 10 days. After 10 days, the seedlings were transplanted into 5 L plastic pots containing the full strength. The composition of the nutrient solution at full concentration was: 1.43 mM NH_4_NO_3_, 1.64 mM MgSO_4_, 0.75 mM CaCl_2_, 0.51 mM K_2_SO_4_, 0.33 mM NaH_2_PO_4_, 20 µM H_3_BO_3_, 10 µM MnCl_2_, 40 µM Fe-NaEDTA, 2.5 µM ZnSO_4_, 0.16 µM CuSO_4_ and 0.08 M (NH_4_)_6_Mo_7_O_2_, 2.5 mM MES buffer modified from previously published studies (Yoshida et al., 1976; Saenchai et al., 2016). The pH was adjusted to 5.5 using hydrochloric acid. The nutrient solution was prepared with deionized water and was renewed every 5 days. Rice seedlings (28 days old) were used for the split-root experiments. The seedlings were transplanted to split-root pots with two compartments each containing 3 liters (L) of nutrient solution for a culture period of 1 week. Iron and Si were supplied as Fe-NaEDTA and K_2_SiO_3_ or omitted from the nutrient solution to impose Fe and Si deficient conditions, abbreviated as –Fe and –Si. 40 µM Fe and 1.5 mM Si were used to prepare the Fe and Si sufficient conditions, abbreviated as +Fe and +Si. Combinations of these conditions were used to prepare the following conditions –Si–Fe/–Si–Fe, –Si–Fe/–Si+Fe, –Si–Fe/+Si–Fe, –Si–Fe/+Si+Fe, –Si+Fe/-Si+Fe, –Si+Fe/+Si+Fe, and +Si+Fe/+Si+Fe in the split-root experiments. Treatment solutions were renewed daily. After 1-week**’**s exposure, roots from different compartments were harvested separately and used to determine Si and Fe concentrations and the transcript accumulation of *Lsi1* using quantitative real-time quantitative reverse-transcription PCR. Experiments were performed in triplicates.

### Real-Time Quantitative Reverse-Transcription PCR

In a split-root system, each part of plant roots were collected separately and immediately frozen in liquid-nitrogen. Next, DNA-free total RNA was extracted from frozen root tissues using Plant RNeasy extraction kit and RNAse-free DNAseI (SIGMA-ALDRCH, St Louis, MO, USA). RNA quality was checked according to Rouached et al. (2008). Total RNA (2µ) were reverse transcribed using ThermoTM script RT (Invitrogen) to obtain the complementary DNA (cDNA). This was used for real-time reverse-transcription PCR, which was performed with LightCycler**^®^**480 (Roche Diagnostics). Care was taken to ensure the specificity for qPCR primers of *Lsi1*. The compositions of PCR reactions were: 12.5 μl of the LightCycler**^®^**480 SYBR Green I Master mix (Roch, IN, USA), forward and reverse primers, and 5 μl of a 1:50 cDNA dilution in a final volume of 25 μl considered for gene expression analysis. Three biological replicates were considered for all PCR reactions. In addition to the one *OsLsi1* gene, a OsActin1 gene was considered for the standardization of real-time PCR data. Quantification of the relative transcripts levels was performed using the comparative CT method as described (Saenchai et al., 2016).

### Iron and Silicon Concentration Measurements

After being harvested, rice roots were collected and then dried at 72°C for 3 days. Then, dried tissues (0.2 g) were ground and subjected to acid digestion. The concentration of Fe in the samples was determined using a Hitachi Z-8230 atomic absorption spectrophotometer (Zarcinas et al., 1987). Si concentration was determined using a spectrophotometer at 650 nm after digestion in 50% NaOH, using the method of Dai et al. (2005). Data are available in **Supplemental Table 1**. Analysis of Si and Fe were based on three independent biological replications.

### Statistical Analysis

ANOVAs analysis was performed using Statistix 8 (analytical software, SXW, Tallahassee, FL, USA). The treatment means comparisons were carried out by least significant difference (LSD) at a probability of p < 0.05.

## Results

### *Lsi1* Is Regulated by Local and Systemic Si Deficiency Signals

**Figure 1:**
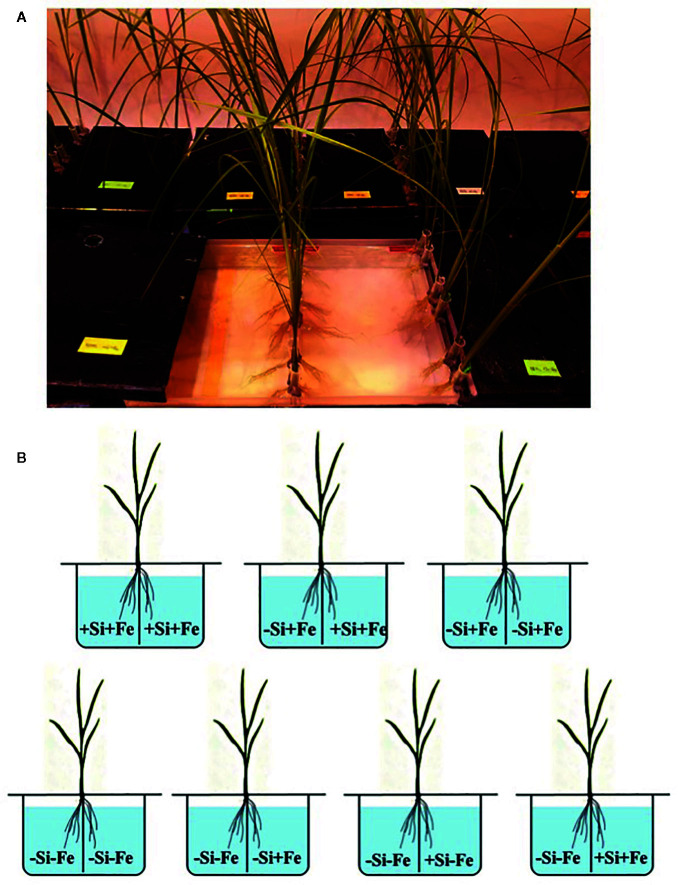
**(A, B)** Treatment combinations for the split-root experiment. The roots of a seedling (28 days old) were split into two parts and immersed in each solution. Iron (Fe) and silicon (Si) were omitted (–Fe and –Si) in the Fe and Si deficient compartment and 40 µM Fe (+Fe) and 1.5 mM Si (+Si) were supplied in the sufficient compartment with seven treatments of +Si+Fe/+Si+Fe, –Si+Fe/+Si+Fe, and –Si+Fe/–Si+Fe, –Si–Fe/–Si–Fe, –Si–Fe/–Si+Fe, –Si–Fe/+Si–Fe and –Si–Fe/ +Si+Fe.

**Figure 2:**
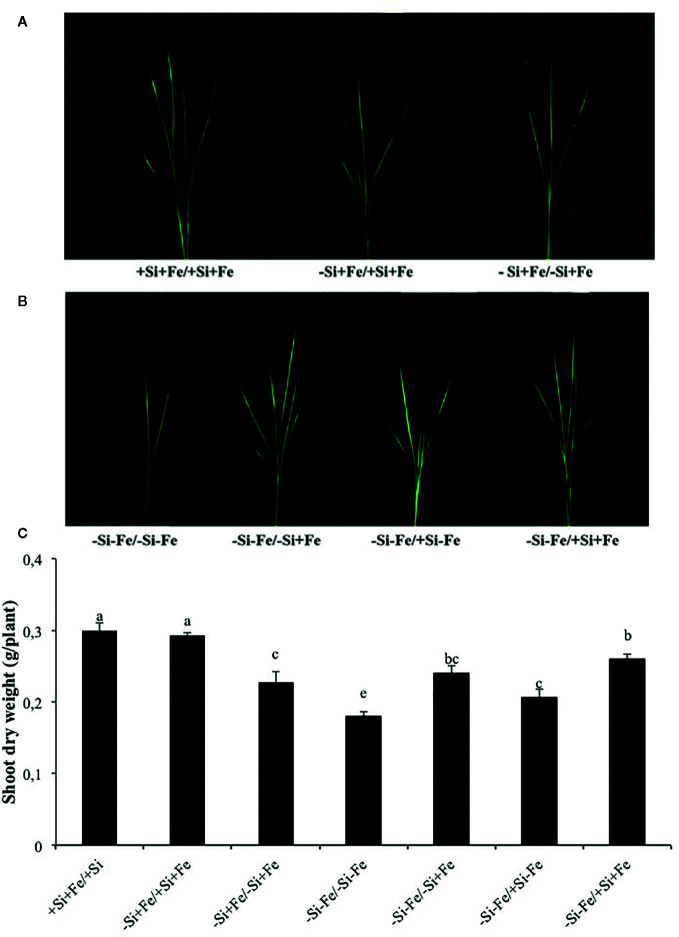
**(A, B)** Phenotypes of rice grown under nutrient solution culture in a split-root system. Si and Fe were supplied with 0 mM Si (–Si) and 0 µM (–Fe), respectively (–Fe and –Si) in the Fe and Si deficient compartment and 40 µM Fe (+Fe) and 1.5 mM Si (+Si) in the sufficient compartment with seven treatments of +Si+Fe/+Si+Fe, –Si+Fe/+Si+Fe, –Si+Fe/-Si+Fe **(A)**, –Si–Fe/–Si–Fe, –Si–Fe/–Si+Fe, –Si–Fe/+Si–Fe, and –Si–Fe/+Si+Fe **(B)** in the split-root experiment. **(C)** Shoot dry weight under aforementioned growth conditions. Analysis was based on three independent biological replicates. Different letters indicate significant differences at P < 0.05

**Figure 3:**
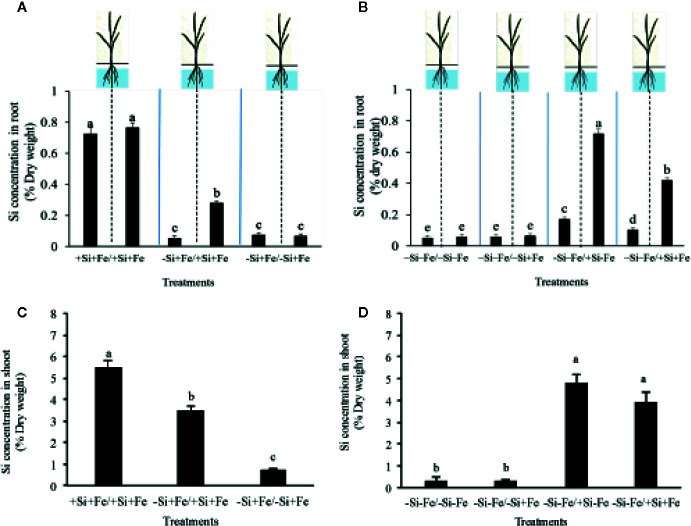
Silicon concentration in root **(A, B)** and shoot **(C, D)** (% dry weight) of PTT1 rice root halves in left or right compartments with nutrient solution culture in a split-root system. Si and Fe were supplied with 0 mM Si (–Si) and 0 µM (–Fe), respectively (–Fe and –Si) in the Fe and Si deficient compartment and 40 µM Fe (+Fe) and 1.5 mM Si (+Si) in the sufficient compartment with seven treatments of +Si+Fe/+Si+Fe, –Si+Fe/+Si+Fe, –Si+Fe/-Si+Fe **(A–C)**, –Si–Fe/–Si–Fe, –Si–Fe/–Si+Fe, –Si–Fe/+Si–Fe, and –Si–Fe/+Si+Fe **(B–D)** in the split-root experiment. Analysis was based on three independent biological replicates. Different letters indicate significant differences at P < 0.05.

**Figure 4:**
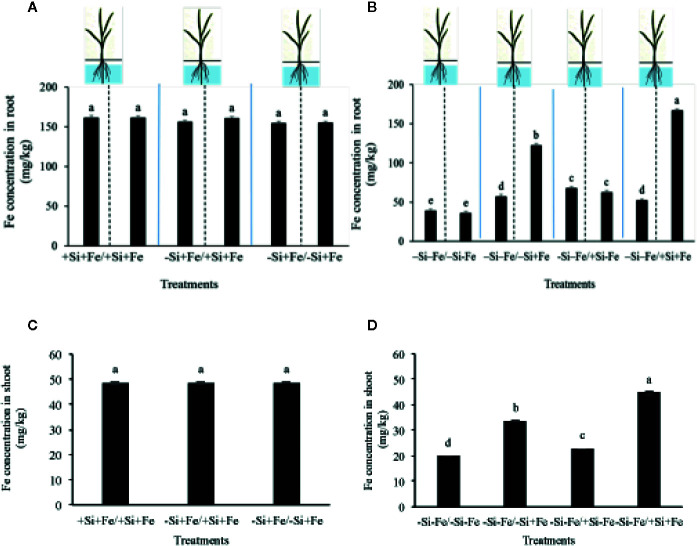
Iron concentration in root **(A, B)** and shoot **(C, D)** (mg/kg dry weight) of PTT1 rice root halves in left or right compartments with nutrient solution culture in a split-root system. Si and Fe were supplied with 0 mM Si (–Si) and 0 µM (–Fe) respectively (–Fe and –Si) in the Fe and Si deficient compartment and 40 µM Fe (+Fe) and 1.5 mM Si (+Si) in the sufficient compartment with seven treatments of +Si+Fe/+Si+Fe, –Si+Fe/+Si+Fe, –Si+Fe/–Si+Fe **(A–C)**, –Si–Fe/–Si–Fe, –Si –Fe/–Si+Fe, –Si–Fe/+Si–Fe, and –Si–Fe/+Si+Fe **(B–D)** in the split-root experiment. Analysis was based on three independent biological replicates. Different letters indicate significant differences at P < 0.05.

**Figure 5:**
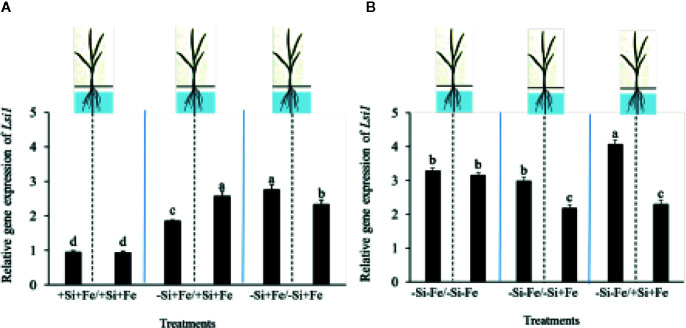
Relative accumulation of Lsi1 transcripts of PTT1 rice root halves in left or right compartments with nutrient solution culture in a split-root system. Si and Fe were supplied with 0 mM Si (–Si), and 0 µM (–Fe), respectively (–Fe and –Si) in the Fe and Si deficient compartment and 40 µM Fe (+Fe) and 1.5 mM Si (+Si) in the sufficient compartment with six treatments of +Si+Fe/+Si+Fe, –Si+Fe/+Si+Fe, –Si+Fe/–Si+Fe, **(A)**, –Si–Fe/–Si–Fe, –Si–Fe/–Si+Fe, and –Si–Fe/+Si+Fe **(B)** in the split-root experiment. Analysis was based on three independent biological replicates. Different letters indicate significant differences at P < 0.05.

While the existence of local and systemic signals regulating nutrient uptake activity has been suggested in many plant species, the existence of a similar mechanism for Si in rice awaits further examination. The present study examines whether the expression of the Si uptake transporter Lsi1 is locally and/or systemically regulated by Si signals in three rice varieties, namely Chainat 1 (CNT1), Pathumthani 1 (PTT1), Khao Dawk Mali 105 (KDML105). These varieties were selected based on their contrasting yield capacity. With regard to Lsi1 expression in response to deficient Si conditions, in order to decipher whether the Si signal was local or systemic, the split-root system was used. In these experiments, the rice root system was divided into two parts, and each part was individually exposed to either Si deficient (–Si) or sufficient conditions (+Si) in the presence of Fe (+Fe) or without Fe (–Fe) (**Figures 1A, B**). Three split root growth conditions were tested: +Si+Fe/+Si+Fe, –Si+Fe/+Si+Fe, –Si+Fe/–Si+Fe. The split-roots exposed to the +Si+Fe/+Si+Fe condition were used as the control of the split-roots exposed to –Si+Fe/+Si+Fe and –Si+Fe/–Si+Fe. First, the concentration of Si and Fe in the roots of plants grown under different conditions was determined. The effects of –Si and/or –Fe on shoot growth was observed, and interestingly, the negative effect of –Si–Fe (–Si–Fe/–Si–Fe) on shoot growth was compensated in the presence of Si on the side of plant roots (+Si–Fe/–Si–Fe) (**Figures 2A, B**). Notably, the responses of Si and Fe were similar among the three rice varieties used in this study (PTT1, **Figures 2A, B**; CNT1, KDML105 **Supplemental Figure 1**). Given the similarity between plant phenotypes, the analysis of the response of PTT1 is given, while the analysis for the CNT1 and KDML105 varieties is included in **Supplemental Data**. It was found that shoot dry weight was decreased by 27% when the whole roots were in the –Si condition (–Si+Fe/–Si+Fe) compared with the shoot when the roots of plants were exposed to +Si+Fe/+Si+Fe treatments. However, no significant difference was observed in the shoots dry weight between +Si+Fe/+Si+Fe (0.30 g/plant) and +Si+Fe/–Si+Fe (0.29 g/plant) conditions (**Figure 2C**, **Supplemental Figures 2** and **3**). In addition, there was no significant difference in root dry weight between the two root halves in all conditions (0.02 g/plant) (data not showed). The results were as expected that –Si conditions decreased Si concentrations in the roots and shoots of plants exposed to –Si+Fe/−Si+Fe compared with the +Si+Fe/+Si+Fe condition. Interestingly, while Si root concentration decreased significantly (92%) when one of the root parts was exposed to –Si treatment, it also decreased (62%) in the root part grown in the presence of +Si treatment (**Figure 3A**, **Supplemental Figures 4** and **5**). Si concentration in shoot of the –Si+Fe/+Si+Fe and –Si+Fe/–Si+Fe were decreased by 36 and 88%, respectively when compared with +Si+Fe/+Si+Fe treatments (**Figure 3C**). However, unlike Si concentrations, no difference was observed for Fe root concentration in the –Si+Fe side (178 mg/kg dry weight) of the split-roots compared to the control roots of +Si+Fe (177 mg/kg dry weight) (**Figure 4A**, **Supplemental Figures 6** and **7**). However, no significant difference was observed in Fe concentration in the shoots (48.5 mg/kg dry weight) (**Figure 4C**). In addition to the phenotype characteristics and physiological analysis, we focused on Lsi1 expression in respect to Si and Fe concentrations in the roots of the split root systems. In line with the previous report for all plants, the experiments in this research revealed that the relative gene accumulation of Lsi1 mRNA increased significantly (approximately two-fold) in the root parts grown in the absence of Si compared to the plant grown in the presence of Si. Nevertheless, it is interesting that despite the presence of Si in one part of the root system, the accumulation of Lsi1 mRNA was found to increase in both parts of the roots exposed to either +Si+Fe or –Si+Fe treatments (**Figure 5A**, **Supplemental Figures 8A** and **9A**). The induction was similar to those observed in plants exposed to –Si+Fe in both sides of split roots (**Figure 5A**, **Supplemental Figures 8** and **9**). This result indicates that Lsi1 is regulated by local Si availability, but also reveals the existence of a long-distance signal to regulate Lsi1.

### Expression of Lsi1 Is Locally Regulated in Response to Si Status and Is Fe-Dependent

As mentioned, there is increasing evidence for an interaction between Si and Fe homeostasis in plants. Therefore, we examined whether the expression of the Si uptake transporter Lsi1 was regulated by Fe-limitation, and how the plant responds to a combined Si and Fe deficiency stresses. The split-root experiments were also carried out with different set of treatments as indicated in **Figures 1 A, B** including –Si–Fe/–Si–Fe, –Si–Fe/–Si+Fe, –Si–Fe/+Si–Fe, and –Si–Fe/+Si+Fe. The split-roots exposed to the –Si–Fe/–Si–Fe condition were used as the control of the split-roots exposed to –Si–Fe/+Si+Fe and –Si–Fe/–Si+Fe and –Si–Fe/+Si–Fe treatments. The concentrations of Si, Fe and Lsi1 mRNA accumulation were measured in each side of all the split-roots. The shoot dry weight in –Si–Fe/+Si–Fe treatment (0.21 g/plant) was significantly higher than that in the –Si–Fe/–Si–Fe (0.18 g/plant) condition but lower than in –Si–Fe/–Si+Fe (0.24 g/plant) and –Si–Fe/+Si+Fe (0.26) treatments. However, no significant difference was observed in dry weights of the shoot between –Si–Fe/–Si+Fe and –Si–Fe/+Si+Fe treatments (**Figure 2C**). The results revealed that stress caused by Fe deficiency had a negative influence on shoot dry weight, but Fe deficiency stress was alleviated when only one root half was supplied with Si. No significant difference in root dry weight was observed between the Si and Fe treatments, with the average of 0.02 g/plant (data not showed). Variations in Si root concentrations in response to Si and Fe deficiency in the split-roots were in agreement with the results obtained in the whole-root experiments. There was no significant difference in the root Si concentration between the two root halves in –Si–Fe/–Si–Fe or –Si–Fe/+Si–Fe. Remarkably, for –Si–Fe/+Si–Fe and –Si–Fe/+Si+Fe treatments, Si concentration in the +Si root half was higher than that in the –Si root half (**Figure 3B**). Indeed, when the half root was exposed to Si (+Si–Fe) and the other half was not (–Si–Fe), the Si concentration in –Si–Fe side was increased compared with plants with both sides exposed to –Si–Fe treatments (**Figure 3B**). This suggests that Si may circulate from the root part that is Si sufficient to the deficient root part. We also noticed that Si concentration in the root exposed to +Si–Fe treatment was higher than Si concentration in the roots exposed to a complete medium (+Si+Fe). This finding indicates that –Fe conditions cause an increase in the Si concentration in roots. Si concentration in the shoot decreased when the whole roots were in the –Si condition (–Si–Fe/–Si–Fe or –Si–Fe/–Si+Fe) compared to the roots of plant exposed to –Si–Fe/+Si–Fe and –Si–Fe/+Si+Fe. In addition, there was no significant difference in shoot Si concentration between the two compartments in –Si–Fe/+Si–Fe or –Si–Fe/+Si+Fe. This suggests that the Si concentration was not affected by the availability of Fe (**Figure 3D**). With regard to Fe concentrations, it was observed that an increase in Fe concentration exposed to –Si+Fe or +Si+Fe when the other part of the root was exposed to –Si–Fe (**Figure 4B**), resulted in –Fe–Si being higher than –Si+Fe. These results further confirm that Fe uptake is regulated by systemic Fe signals, as well as the integration of signals between Si and Fe. In addition, shoot Fe concentration was increased when half of roots were in the +Si or +Fe condition. Interestingly, shoot Fe concentration was increased by 14% when grown in the –Si-Fe/+Si-Fe treatment compared to the –Si-Fe/-Si-Fe treatment (**Figure 4D**). Finally, we determined the relative transcript accumulation of Lsi1. The results showed that Lsi1 was up regulated by –Si stress when plants were subjected to –Si–Fe in both sides of the root system. Nevertheless, regardless of the absence of Si in the medium, the presence of Fe in the root sides attenuates the induction of Lsi1 expression by –Si (**Figure 5B**).

## Discussion

This study provides new insight into how plants integrate Si and Fe signals to regulate Si concentrations, suggesting a possible translocation between the two parts of plant roots affecting on the shoot growth of rice plants. Applying Si enhanced shoot growth as shown by increasing of the dry weight compared with Si deficiency, which has also been reported (Kim et al., 2012). The current results further demonstrated the beneficial effect of Si under Fe deficiency stress on shoot growth of rice. It was found that under Fe deficiency the rice plants grown without Si were severely chlorotic, while in Si-supplied plants chlorosis was alleviated. Applying Si was found to alleviate Fe deficiency symptoms in cucumber plants by increasing shoot and root biomass and chlorophyll content attributed to the enhancement of the mobilization of Fe in the root and increased Fe uptake and translocation under deficiency conditions (You-Qiang et al., 2012). In addition, the increase in shoot growth under Fe deficiency might be due to difference of Si concentration in the shoot. Gonzalo et al. (2013) reported that the increase of Si content in shoot as a result of Si application could increase leaf photosynthetic activity and prevent the destruction of chlorophyll under Fe deficiency. Our split-root experiments revealed that Si concentration in the +Si+Fe root half in the –Si+Fe/+Si+Fe condition decreased when other root parts were not exposed to Si compared with +Si+Fe/+Si+Fe roots, indicating that Si starvation on one side of the root system leads to a decrease as well in the concentration on the other side. A similar observation in phosphorus (P) showed that the concentration of P in the sufficient root parts was decreased when the other root half limited P supply compared to the roots exposed to P in the whole roots which indicates that the extent of P concentration in roots might depend on the external P supply (Shane et al., 2003; Shen et al., 2004). In addition, nitrogen (N) starvation on one side of the roots affected on N uptake in other parts of the roots exposed to N supply conditions in a heterogeneous N condition (O’Brien et al., 2016). Under Fe deficiency, the external Si supply to one root half significantly increased the Si concentration in Si-deficient parts of the roots compared with plants with both sides exposed to –Si–Fe treatments. This was probably due to Si-deficient parts of the roots being increased by the Si sufficient root parts which is consistent with the findings of a previous study where a –P root half significantly increased when the other root half received P supply in maize and lupin due to P translocation from the P sufficient part to the deficient part ***via*** the shoot (Shen et al., 2004). Also, a long-distance signaling pathway for P and N homeostasis has been reported (Chiou and Lin, 2011; Tabata et al., 2014). The results in this study are probably related to the expression of Si transporter genes, *OsLsi1* and *OsLsi2* in the roots as reported recently in the split-root study that Si starvation on one side of the roots induced changes of *OsLsi1* and *OsLsi2* expression in the roots (Mitani-Ueno et al., 2016). A possible explanation that may account for the observation of high levels of Si in the −Si side of the split-roots is that the Si translocated from Si sufficient parts of the plant in the −Si side of roots, which could contribute to maintain the high levels of Si in the −Si side of the root. This process appears to be independent of the Fe availability in the medium because there was compensation of Si concentration in –Si–Fe parts of the root by the +Si–Fe or +Si+Fe parts. Such an explanation should be examined with others Si transporters such as *Lsi2*. Our results strongly argue against a relay model in which Si would be a significant intermediary in the induction of the expression of *Lsi1*, even if it may be a component of this regulation.

Available data revealed that the presence of complex coordination among the mineral nutrient-derived signals is the rule rather than the exception. Nevertheless, the interdependency of nutrient signals has not been well characterized to date (Bouain et al., 2019). Using split root experiments, our study demonstrated that the root Si uptake system involved in the response to Si deficiency is controlled by both local and systemic signals. First, Si itself seems to act locally as an inducer because Si deficiency promotes *Lsi1* transcripts. Second, *Lsi1* is also under the control of a systemic regulation that occurs in Si deficiency conditions for at least two reasons. *Lsi1* is induced in Si sufficient roots when one part of the roots is subjected to –Si stress. Furthermore, our results show for the first time that this long distance signal affects the response of silicon *Lsi1* expression, which is influenced by the availability of Fe. Moreover, the regulation of genes involved in nutrient uptake transport can involve both local and systemic signals as has been reported (S, N, Fe, etc.…) (Rouached et al., 2008; Saenchai et al., 2016), but the interdependency of nutrient signals such +Si–Fe is new. In light of these new results and the existing data, it is clear that the regulation of mineral nutrient homeostasis, as previously postulated, involves each individual nutrient level being controlled by its own nutrient-specific mechanisms and signaling pathways. Our results demonstrate that shoot growth is involved in regulating the Si deficiency response in the root. The nature and identity of the signal deserves further investigation. Finally, because the responses to Si and Fe single and combined stresses between the three rice cultivars analyzed in the context of this work were similar, we are tempted to propose that the mechanisms that regulate Si and Fe signaling pathways are maintained within plant species. Whether they are maintained during evolution between plant species needs further investigation, which will be feasible with the development of systemic approaches in different monocots and/or dicot plants.

## Conclusion

Our study has showed the regulation of the expression of Si uptake transporter *Lsi1* in rice. It has established that the expression of *Lsi1* is dependent on the local root Si concentration and is influenced by Fe availability. Our results further reinforce the general schema in which there is an obvious role for shoot-to-root signaling to regulate nutrient uptake and expression of the mineral transporters in rice plants. Understanding the molecular basis of these Fe and Si signals**’** crosstalk is seen as an essential step towards developing novel strategies to create rice varieties with the capacity to adapt well to their environment and increase productivity.

## Data Availability Statement

The raw data supporting the conclusions of this article will be made available by the authors, without undue reservation.

## Author Contributions

HR and CP-U-T designed the research. HR supervised this project. NC and NB performed most experiments. HR and CP-U-T analyzed the data and wrote the manuscript. All authors contributed to the article and approved the submitted version.

## Funding

This work was funded by the Institut National de la Recherche Agronomique (INRAE), and in part by Michigan State University (USA) to HR, a Royal Golden Jubilee Ph.D. Program (Grant No. PHD/0153/2557) to NC, and partially supported by Chiang Mai University, Thailand.

## Conflict of Interest

The authors declare that the research was conducted in the absence of any commercial or financial relationships that could be construed as a potential conflict of interest.
