# Supplementary material for: Interplay Between Silicon and Iron Signaling Pathways to Regulate Silicon Transporter Lsi1 Expression in Rice
Source: Front Plant Sci. 2020 Jul 22;11:1065. doi: 10.3389/fpls.2020.01065 (PMC7387502; doi:10.3389/fpls.2020.01065)
Supplement: Supplementary file 1 [file DataSheet_1.pdf]

Supplementary Fig. 1

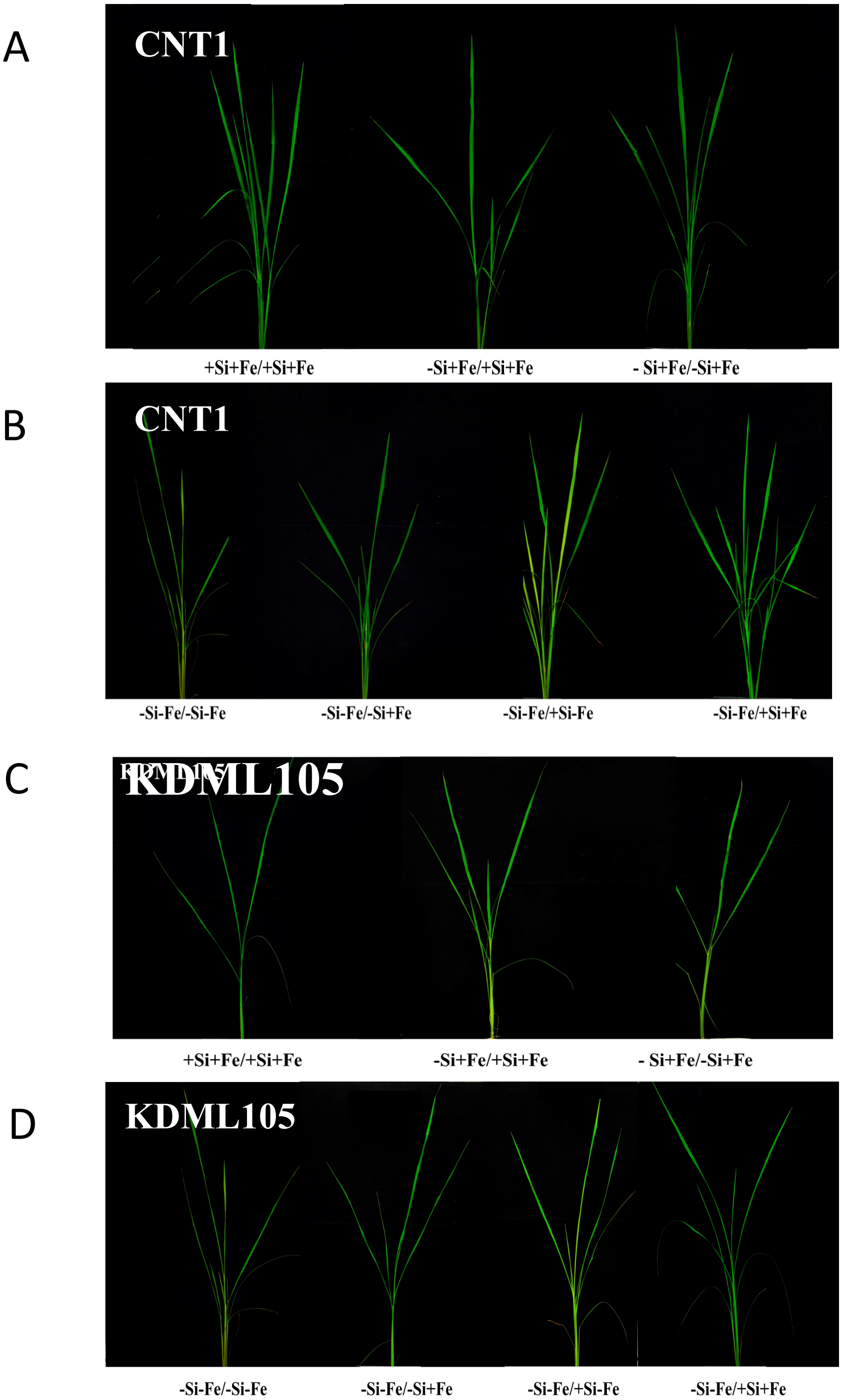

Supplemental Fig. 1 A-B) Phenotypes of CNT1 rice grown under nutrient solution culture in a split-root system. Si and Fe were supplied with 0 mM Si (–Si) and 0 µM (–Fe) respectively (–Fe and–Si) in the Fe and Si-deficient compartment and 40 µM Fe (+Fe) and 1.5 mM Si (+Si) in the sufficient compartment with seven treatments of +Si+Fe/+Si +Fe, –Si+Fe/+Si+Fe, –Si+Fe/–Si+Fe (B), –Si–Fe/–Si–Fe, –Si–Fe/–Si+Fe, –Si–Fe/+Si–Fe, and –Si–Fe/+Si+Fe (B) in the split-root experiment**. C-D)** Phenotypes of KDML105 rice grown under nutrient solution culture in a split-root system. Si and Fe were supplied with 0 mM Si (–Si) and 0 µM (–Fe) respectively (–Fe and –Si) in the Fe and Si deficient compartment and 40 µM Fe (+Fe) and 1.5 mM Si (+Si) in the sufficient compartment with seven treatments of +Si+Fe/+Si+Fe, –Si+Fe/+Si+Fe, –Si+Fe/–Si+Fe, –Si–Fe/–Si–Fe, –Si–Fe/–Si+Fe, –Si–Fe/+Si–Fe, and –Si–Fe/+Si+Fe in the split-root experiment**.**

Supplementary Fig. 2

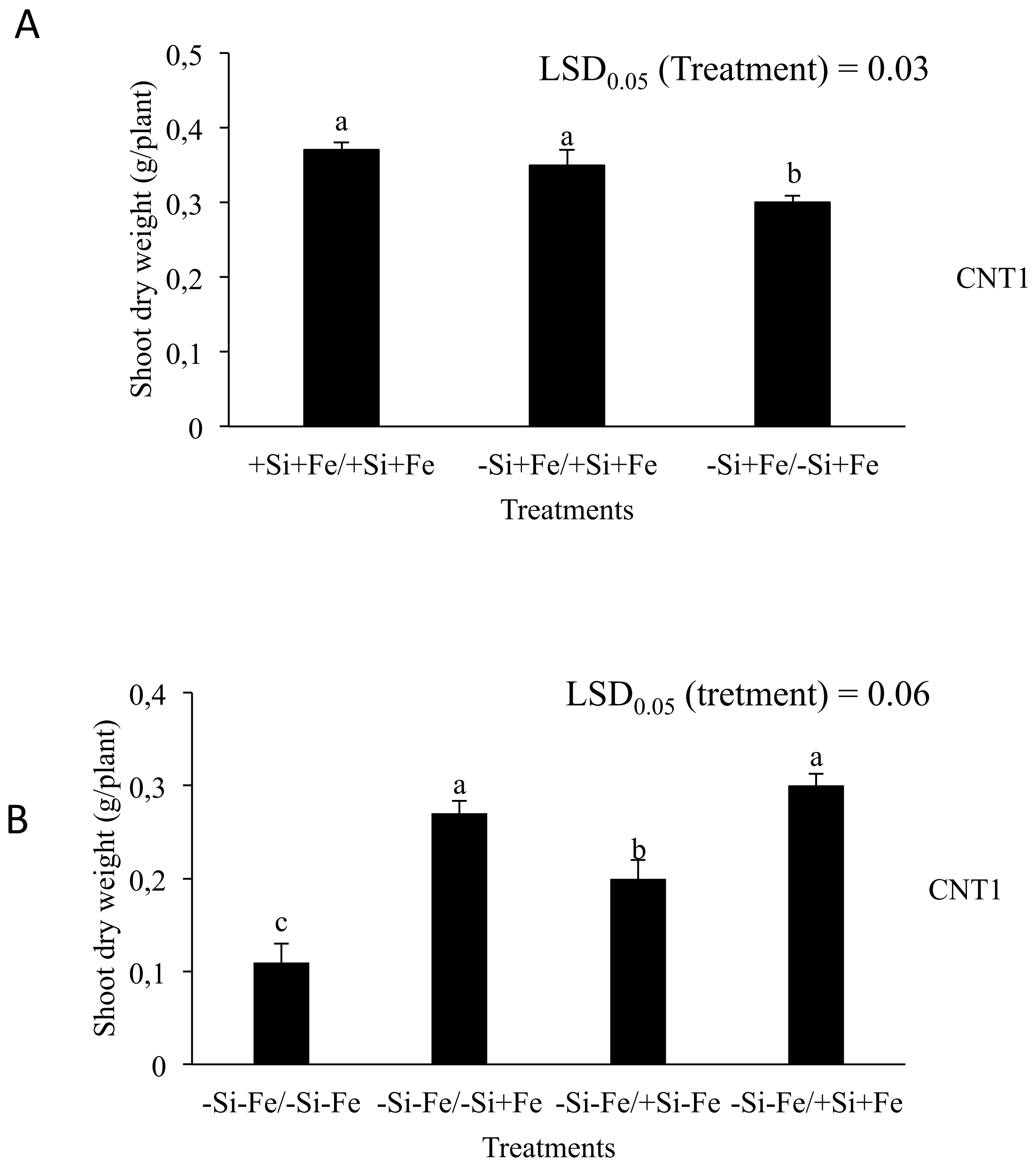

Supplemental Fig. 2 A-B). Shoot dry weight of CNT1 grown under different Fe and Si regimes.

Supplementary Fig. 3

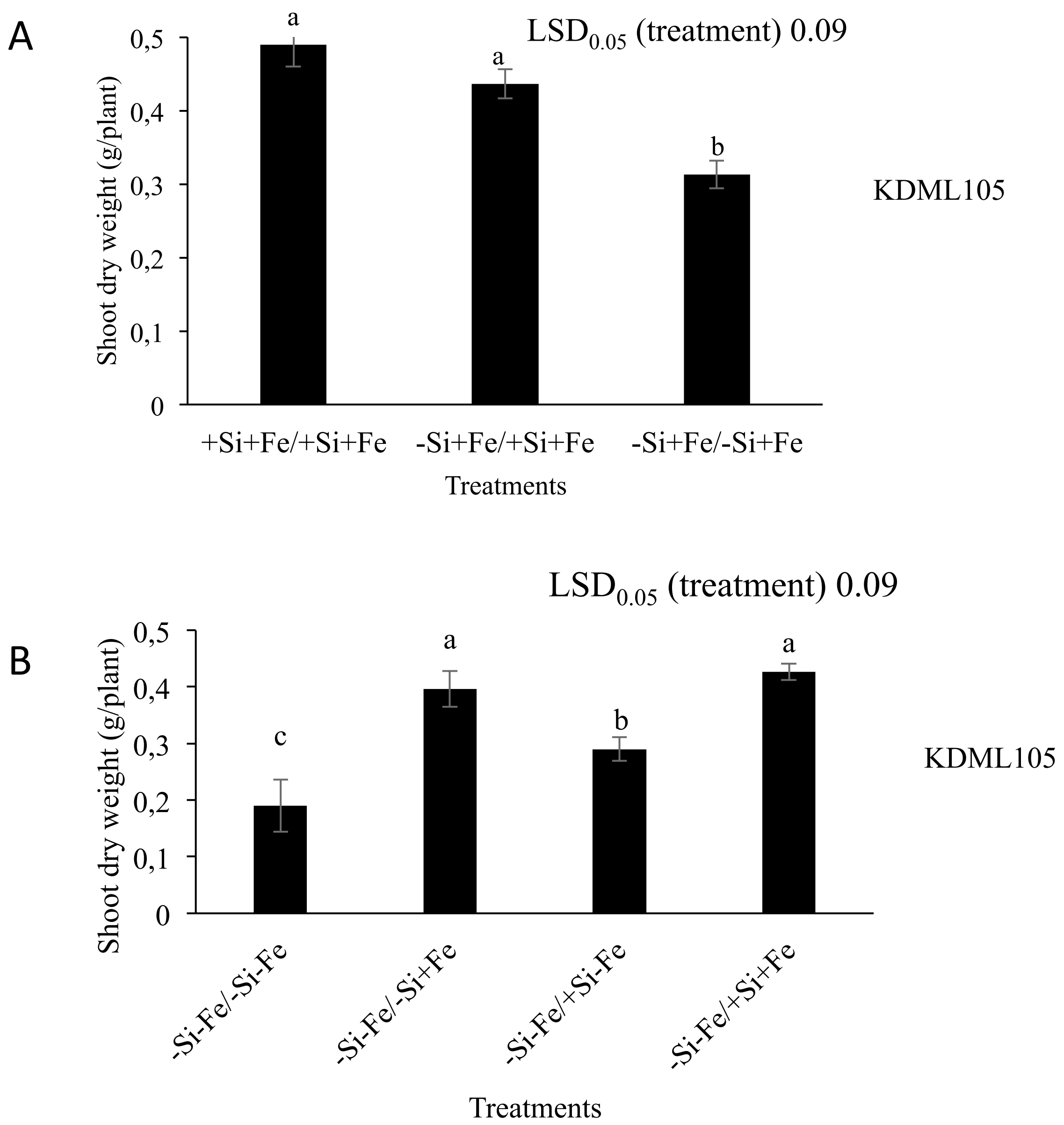

Supplemental Fig. 3 A-B). Shoot dry weight of KDML105 grown under different Fe and Si regimes.

Supplementary Fig. 4

A

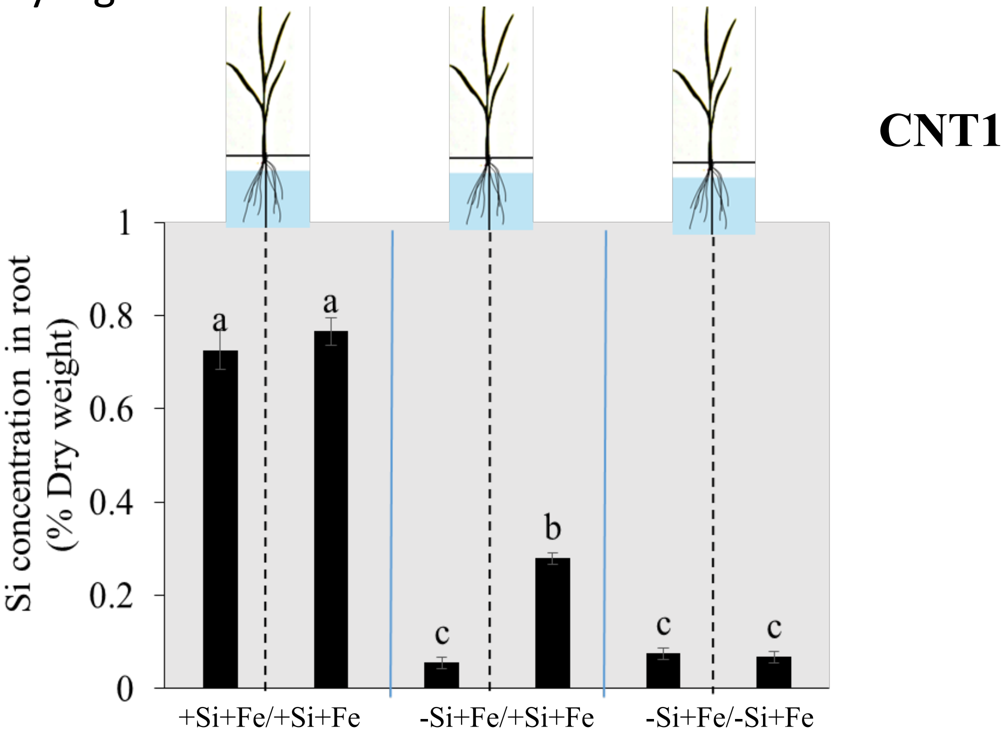

B

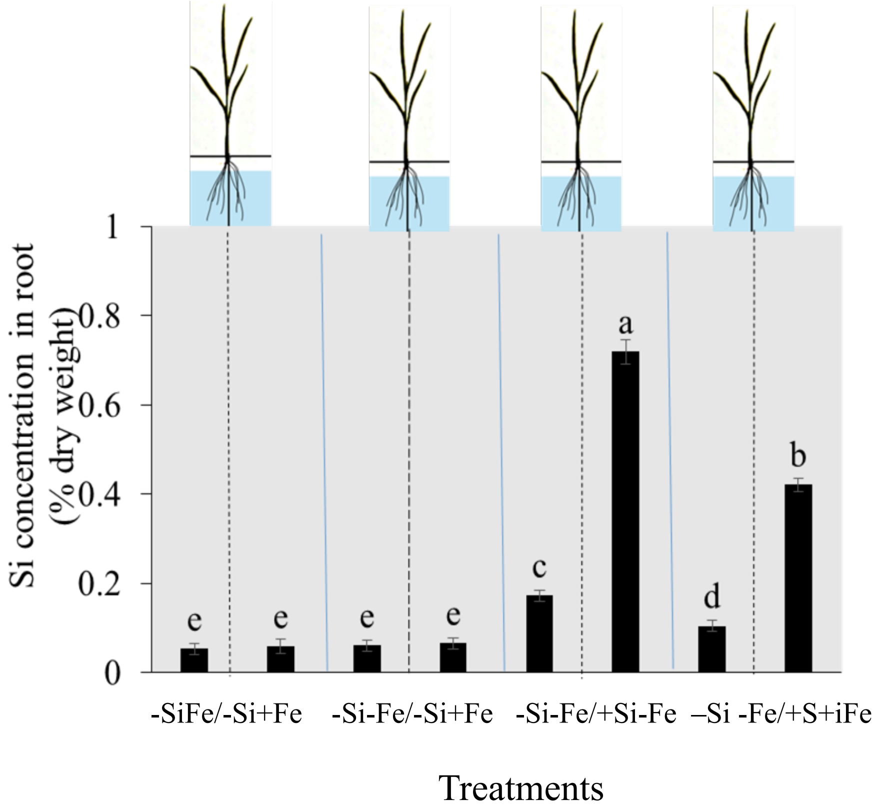

Supplemental Fig. 4 Silicon (Si) concentration in root (mg/kg dry weight) of CNT1 rice root halves in left or right compartments with nutrient solution culture in a split-root system. Si and Fe were supplied with 0 mM Si (-Si) and 0 µM (-Fe) respectively (-Fe anfd-Si) in the Fe and Si-deficient compartment and 40 µM Fe (+Fe) and 1.5 mM Si (+Si) in the sufficient compartment with seven treatments of +Si+Fe/+Si+Fe, -Si+Fe/+Si+Fe, -Si+Fe/-Si+Fe (A), -Si-Fe/-Si-Fe, -Si -Fe/-Si+Fe, -Si-Fe/+Si-Fe, and -Si-Fe/+Si+Fe (B) in the split-root experiment.

Supplementary Fig. 5

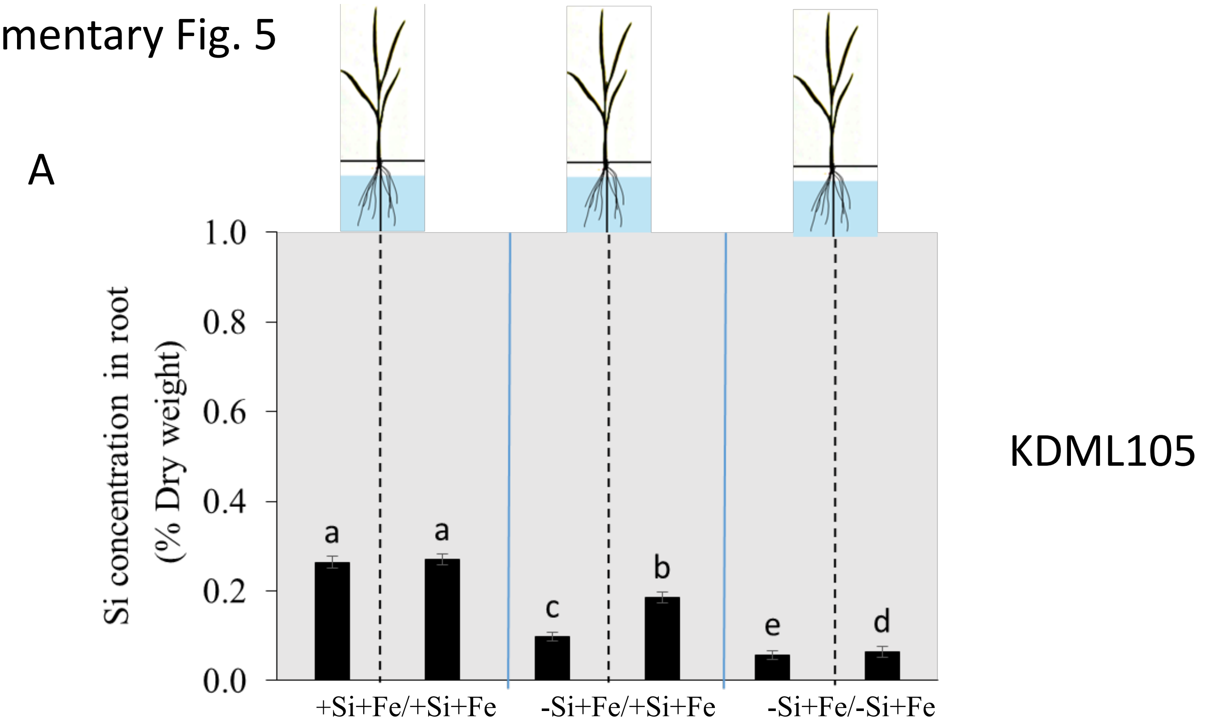

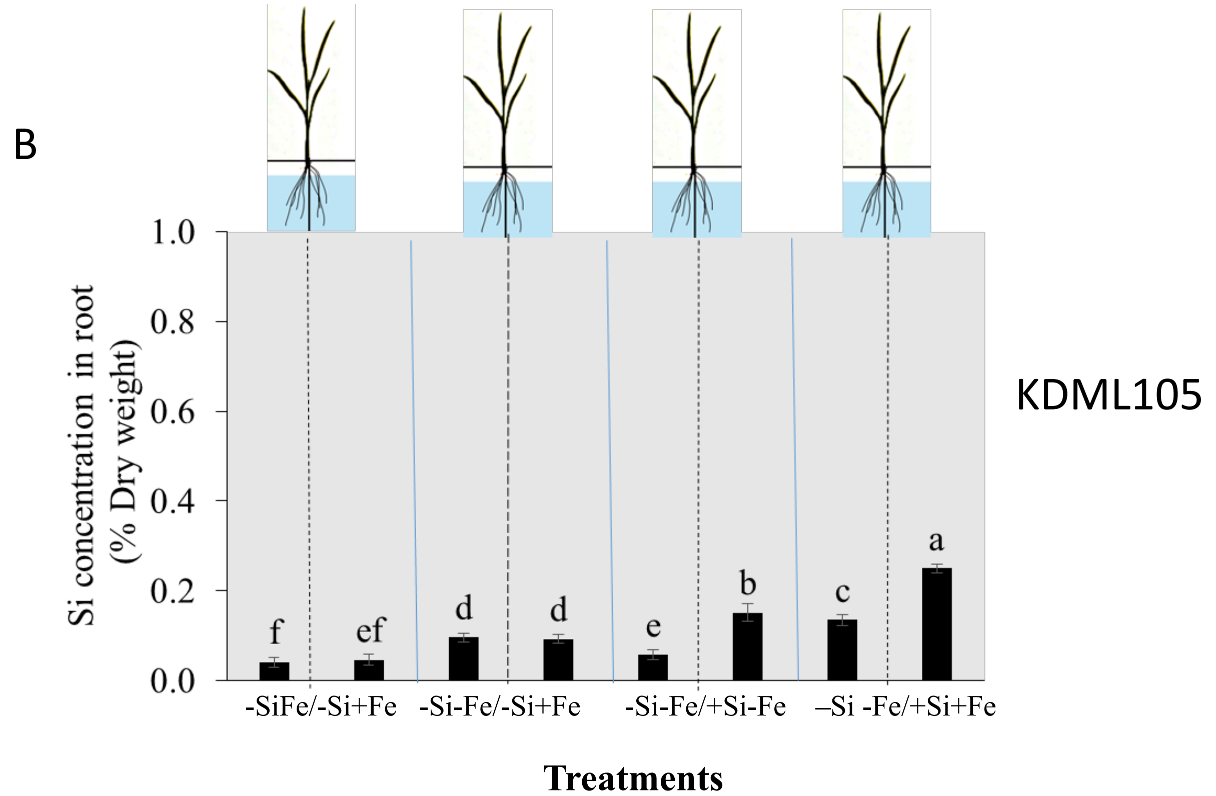

Supplemental Fig. 5 Silicon (Si) concentration in root (mg/kg dry weight) of KDML105 rice root halves in left or right compartments with nutrient solution culture in a split-root system. Si and Fe were supplied with 0 mM Si (-Si) and 0 µM (-Fe) respectively (-Fe anfd-Si) in the Fe and Si-deficient compartment and 40 µM Fe (+Fe) and 1.5 mM Si (+Si) in the sufficient compartment with seven treatments of +Si+Fe/+Si+Fe, -Si+Fe/+Si+Fe, -Si+Fe/-Si +Fe (A), -Si-Fe/-Si-Fe, -Si -Fe/-Si+Fe, -Si-Fe/+Si-Fe, and -Si-Fe/+Si+Fe (B) in the split-root experiment.

Supplementary Fig. 6

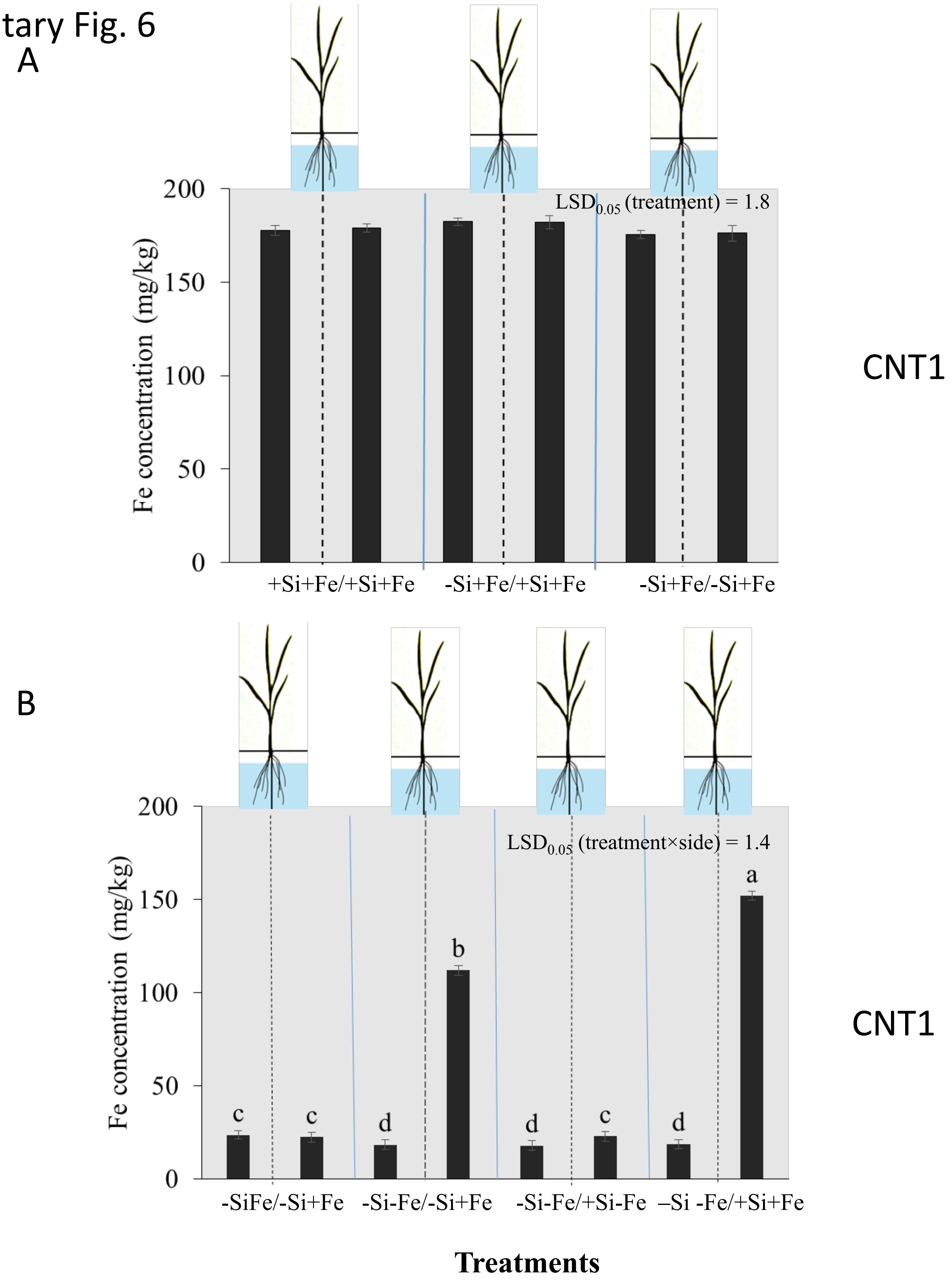

Supplemental Fig. 6 Iron (Fe) concentration in root (mg/kg dry weight) of CNT1 rice root halves in left or right compartments with nutrient solution culture in a split-root system. Si and Fe were supplied with 0 mM Si (-Si) and 0 µM (-Fe) respectively (-Fe anfd-Si) in the Fe and Si-deficient compartment and 40 µM Fe (+Fe) and 1.5 mM Si (+Si) in the sufficient compartment with seven treatments of +Si+Fe/+Si+Fe, -Si+Fe/+Si+Fe, -Si+Fe/-Si+Fe (A), -Si-Fe/-Si-Fe, -Si -Fe/-Si+Fe, -Si-Fe/+Si-Fe, and -Si-Fe/+Si+Fe (B) in the split-root experiment.

Supplementary Fig. 7

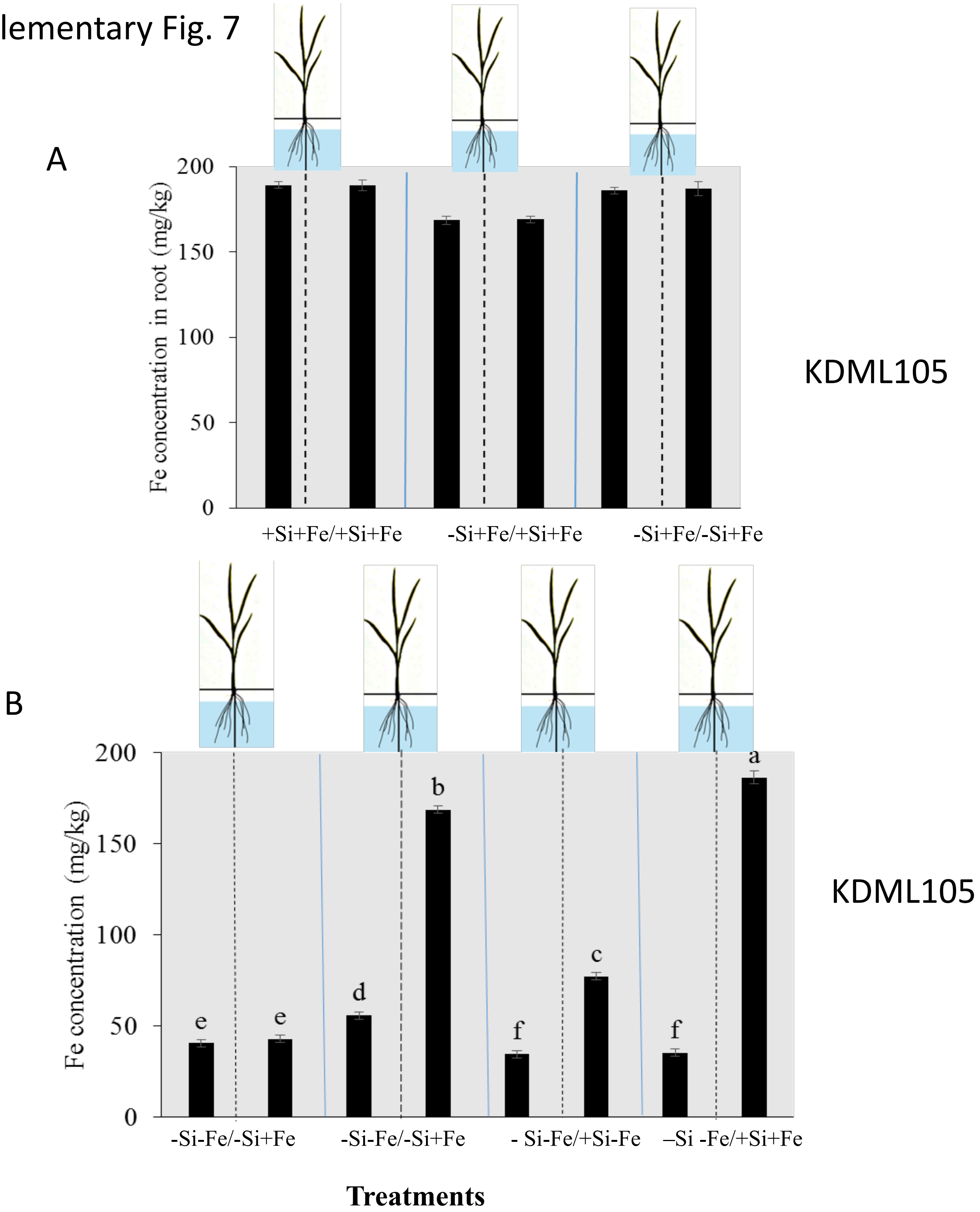

Supplemental Fig. 7 Iron (Fe) concentration in root (mg/kg dry weight) of KDML105 rice root halves in left or right compartments with nutrient solution culture in a split-root system. Si and Fe were supplied with 0 mM Si (-Si) and 0 µM (-Fe) respectively (-Fe anfd-Si) in the Fe and Si-deficient compartment and 40 µM Fe (+Fe) and 1.5 mM Si (+Si) in the sufficient compartment with seven treatments of +Si+Fe/+Si+Fe, -Si+Fe/+Si+Fe, -Si+Fe/-Si +Fe (A), -Si-Fe/-Si-Fe, -Si -Fe/-Si+Fe, -Si-Fe/+Si-Fe, and -Si-Fe/+Si+Fe (B) in the split-root experiment.

Supplementary Fig. 8

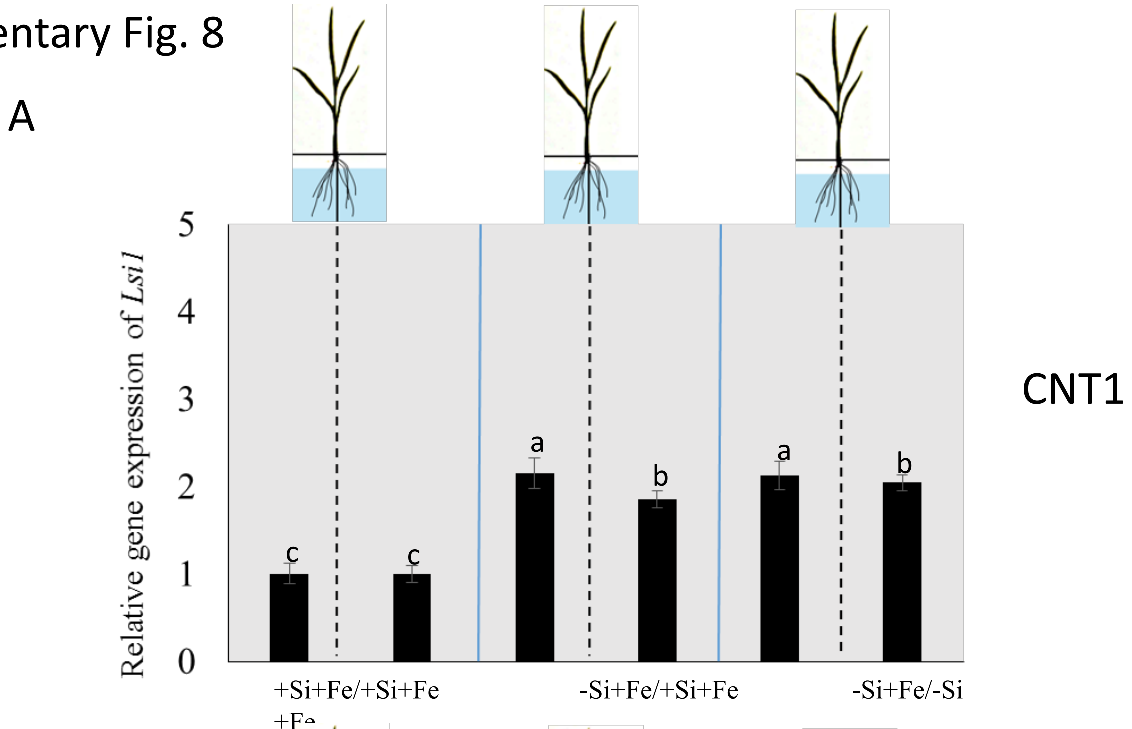

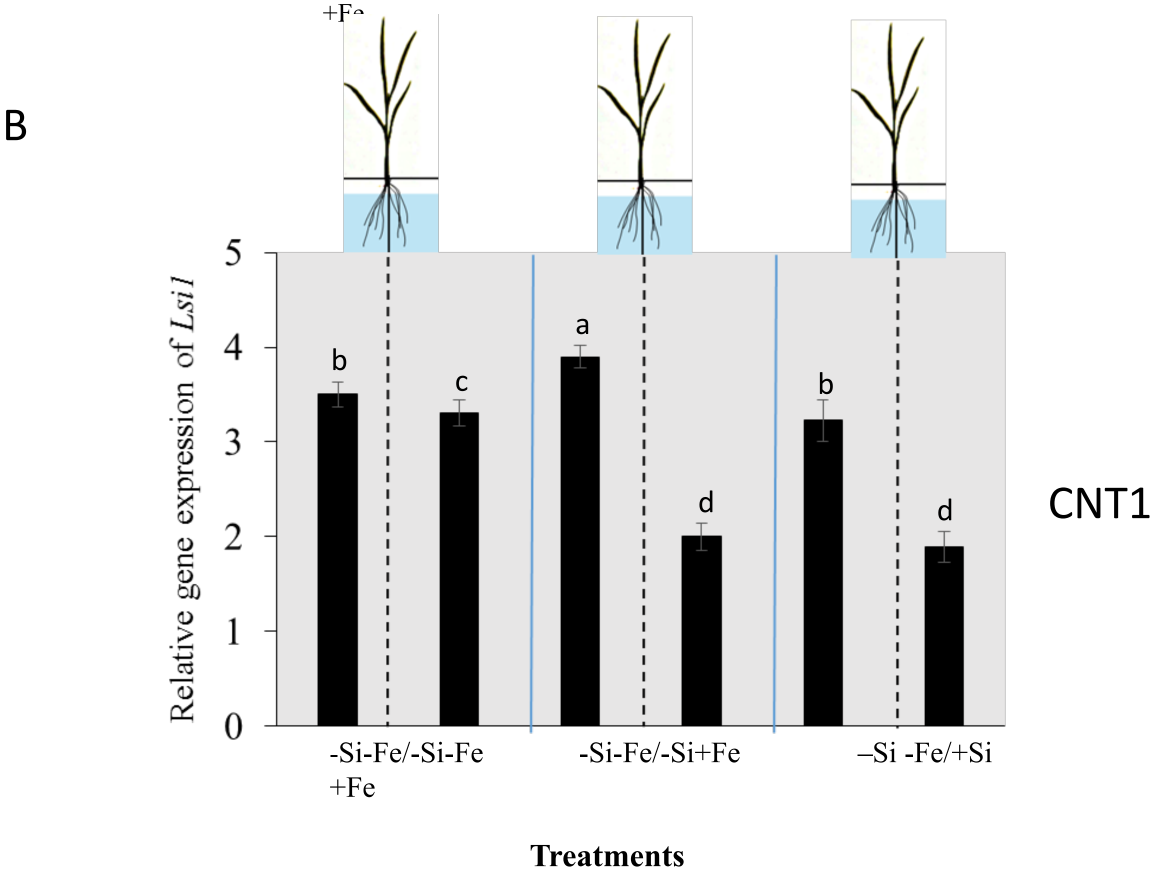

Supplemental Fig. 8 Relative accumulation of *LSi1* mRNA of CNT1 rice root halves in left or right compartments with nutrient solution culture in a split-root system. Si and Fe were supplied with 0 mM Si (-Si), and 0 µM (-Fe) respectively (-Fe anfd-Si) in the Fe and Si-deficient compartment and 40 µM Fe (+Fe) and 1.5 mM Si (+Si) in the sufficient compartment with seven treatments of-+Si+Fe/+Si+Fe, -Si+Fe/+Si+Fe, -Si+Fe/-Si+Fe, (A), -Si-Fe/-Si-Fe, -Si-Fe/-Si+Fe, -Si-Fe/+Si-Fe, and -Si-Fe/+Si+Fe (B) in the split-root experiment.

Supplementary Fig. 9

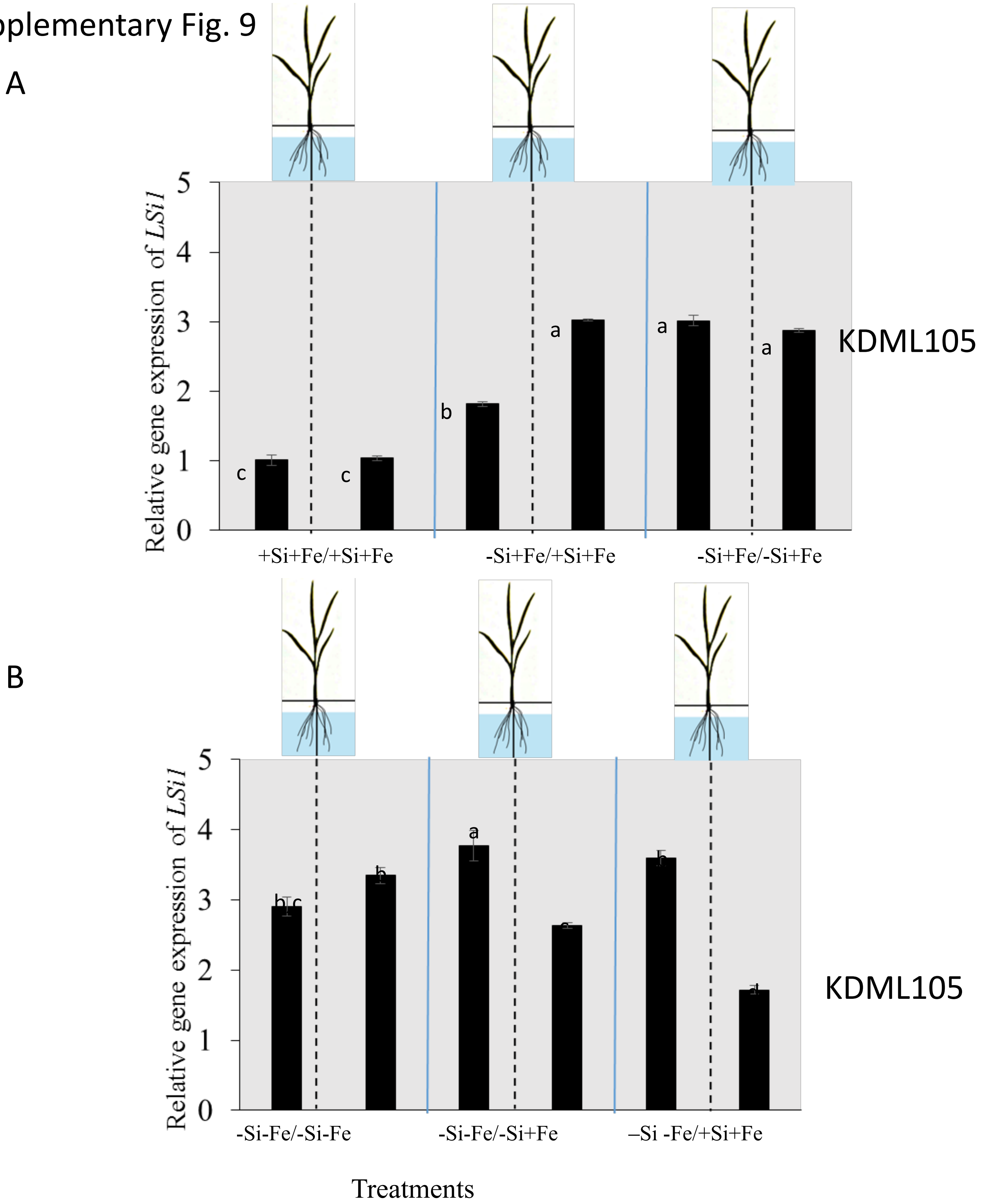

Supplemental Fig.9 Relative accumulation of LSi1 mRNA of KDML105 rice root halves in left or right compartments with nutrient solution culture in a split-root system. Si and Fe were supplied with 0 mM Si (-Si), and 0 µM (-Fe) respectively (-Fe anfd-Si) in the Fe and Si-deficient compartment and 40 µM Fe (+Fe) and 1.5 mM Si (+Si) in the sufficient compartment with seven treatments of-+Si+Fe/+Si+Fe, -Si+Fe/+Si+Fe, -Si+Fe/-Si+Fe, (A), -Si-Fe/-Si-Fe, -Si-Fe/-Si+Fe, -Si-Fe/+Si-Fe, and -Si-Fe/+Si+Fe (B) in the split-root experiment.
